# Artificial Intelligence in Addressing Interpersonal Violence Against Vulnerable Populations: A Scoping Review

**DOI:** 10.1590/0034-7167-2025-0547

**Published:** 2026-08-21

**Authors:** Hallessa de Fátima da Silva Pimentel, Julia Sunmy Aguni Chunga, Ana Maria José Garcia João Pascoal, Dulce Aparecida Barbosa, Claudia Galindo Novoa, Hugo Fernandes

**Affiliations:** I Universidade Federal de São Paulo. São Paulo, São Paulo, Brazil; II Universidade Agostinho Neto. Talatona, Angola

**Keywords:** Artificial Intelligence, Violence, Vulnerable Populations, Computer Systems, Public Health., Inteligencia Artificial, Violencia, Poblaciones Vulnerables, Sistemas de Computación, Salud Pública.

## Abstract

**Objectives:**

to map and summarize the main applications of artificial intelligence in addressing interpersonal violence against vulnerable populations.

**Methods:**

a scoping review was conducted between December 2024 and February 2025 in the PubMed, Web of Science, Embase, PsycINFO, and CINAHL databases, as well as grey matter databases. Editorials, retractions, and studies of self-inflicted violence were excluded.

**Results:**

750 texts were found. After applying the eligibility criteria, a sample of ten materials was obtained. The studies were grouped into the categories “Prediction and automated monitoring of violence”, “Decision support, technological innovation in health and social protection” and “Ethics, security, and social impact of artificial intelligence in vulnerable contexts”.

**Conclusions:**

artificial intelligence models for addressing violence against vulnerable populations require verification, data protection, social governance and testing with a high level of evidence.

## RESUMEN

**Objetivos:**

mapear y resumir las principales aplicaciones de la inteligencia artificial para abordar la violencia interpersonal contra poblaciones vulnerables.

**Métodos:**

se realizó una revisión exploratoria entre diciembre de 2024 y febrero de 2025 en las bases de datos PubMed, *Web of Science*, Embase, PsycINFO y CINAHL, así como en bases de datos de materia gris. Se excluyeron editoriales, retractaciones y estudios sobre violencia autoinfligida.

**Resultados:**

se encontraron 750 textos. Tras aplicar los criterios de elegibilidad, se obtuvo una muestra de diez materiales. Los estudios se agruparon en las categorías “Predicción y monitoreo automatizado de la violencia”, “Apoyo a la toma de decisiones, innovación tecnológica en salud y protección social” y “Ética, seguridad e impacto social de la inteligencia artificial en contextos vulnerables”.

**Conclusiones:**

los modelos de inteligencia artificial para abordar la violencia contra poblaciones vulnerables requieren verificación, protección de datos, gobernanza social y pruebas con un alto nivel de evidencia.

## INTRODUCTION

Interpersonal violence is any intentional action that causes harm to another person, whether physical, psychological, sexual, moral, or financial. This violence can occur in various contexts, such as family, school, work, and community, and involves the use of force, power, threats, or other forms of coercion to control, dominate, or harm someone. It is a complex health problem with diverse causes and consequences, and social inequality, sexism, racism, and other types of discrimination can contribute to its increase^(1)^.

There can be a feedback loop involving violence and social vulnerability. Individuals experiencing poverty, social inequality, discrimination, or exclusion are significantly more susceptible to becoming victims of violence. Social and economic class, for instance, can limit access to basic resources such as education, health, and security, making people more vulnerable to various forms of violence^(2)^. Furthermore, social inequality creates an environment conducive to discrimination and exclusion, marginalizing specific groups such as women, black people, older adults, children, people from the lesbian, gay, bisexual, transgender, queer, intersex, asexual, and other sexual minority communities (LGBTQIA+), as well as people experiencing homelessness. This marginalization makes them easy targets for aggressors, who find in the vulnerability of these people an opportunity to exercise power and domination^(2,3)^. The intersectionality of social vulnerability, i.e., the combination of different social markers such as race, gender, class, and sexual orientation, further aggravates this situation, exposing these people to multiple forms of oppression and violence.

The Pan American Health Organization estimates that, among women alone, one in three women between the ages of 15 and 49 has suffered physical and/or sexual violence, and among minors under 15, one in four has suffered the same abuse, with family members or acquaintances being the main aggressors^(4)^. Brazilian researchers state that older adults also have a high incidence of interpersonal violence perpetrated by family members, but that the most common types include psychological or moral and financial abuse^(5)^. Regarding the LGBTQIA+ community, scholars point out that transgender people have their human rights severely violated, constantly being targets of physical, moral, and sexual aggression^(3)^.

Australian researchers cite that healthcare professionals still face significant challenges in making quick and assertive decisions when caring for victims of violence, especially those with significant underlying social determinants^(6)^. Even when there are clear and well-established protocols, healthcare professionals responsible for initial care do not always know which actions to prioritize and what referrals should be made to reduce harm to victims and witnesses. The reasons can range from insufficient training on care pathways and/or lack of empathy to difficulties in prioritizing where to send these individuals, such as specialized police stations, protection services, and others^(7,8)^.

A more recent technology that appears promising for reducing errors and improving care for victims in healthcare services, especially in complex cases or those requiring very quick decisions from healthcare professionals, is artificial intelligence (AI). Researchers have been experimenting with the use of neural networks, machine training, and language models as a tool to help address this reality^(9,10)^. However, since it is a relatively new resource in the healthcare field, its implementation may involve special precautions, such as the confidential storage of data from a case of violence.

Searches on the Open Science Framework and International Prospective Register of Systematic Reviews platforms yielded no records of systematic and/or scoping reviews on the topic, potentially indicating a novelty that motivates the development of this investigation. Thus, the relevance of this research lies in compiling evidence on addressing interpersonal violence against populations with greater social vulnerability, mediated by AI.

## OBJECTIVES

To map and summarize the main applications of AI in addressing interpersonal violence against vulnerable populations.

## METHODS

### Ethical aspects of research

National and international legislation regarding ethics in research involving human subjects was fully respected. Due to the nature of the research, which did not involve direct contact with people or animals, submission to and approval by a Research Ethics Committee was waived. However, a declaration of responsibility and assessment by the Department of Public Health was completed, in accordance with Resolution 00 of 2021 of the *Universidade Federal de São Paulo*’s University Council.

### Study design and period

This is a scoping literature review, structured according to JBI recommendations^(11)^, which were: 1) initial exploratory search to find primary references, which are records of studies that met the inclusion criteria for the review question; 2) following a comprehensive search strategy using key indexed descriptors from the protocol; and 3) supplementary grey literature search, which included citation search and manual search.

Data collection was conducted between December 2024 and February 2025. Searches were performed in the National Library of Medicine (PubMed), Web of Science, Excerpta Medica DataBASE (Embase), PsycINFO - APA PsycNET (American Psychological Association), and Cumulative Index to Nursing and Allied Health Literature (CINAHL) databases. Gray references in publicly and unrestricted access theses and dissertations were searched in MedNar, WorldWideScience, and Google Scholar. Additional studies were also included from references in the primary articles (manual search).

### Inclusion and exclusion criteria

Studies on AI and interpersonal violence against vulnerable populations (women, children, older adults, homeless people, black people, and members of the LGBTQIA+ community), published in Portuguese, Spanish, or English, without a time frame, were included. Editorials, retractions, and studies focused on self-inflicted violence or suicide attempts were excluded. The level of evidence was not considered an exclusion criterion, justifying this as it is still a recent topic in the health field. The review report was prepared according to the Preferred Reporting Items for Systematic Reviews and Meta-Analyses Extension for Scoping Reviews: Checklist and Explanation^(12)^.

### Study protocol

To develop the review question, the following phases were followed: question identification; search for relevant studies; data selection and extraction; and results grouping, summary, and presentation. The PCC (Population, Concept, and Context) strategy was adopted for formulating the research question with the following representations: P = vulnerable populations (women, children, older adults, people experiencing homelessness, black people, and members of the LGBTQIA+ community) victims of violence; C = AI; and C = healthcare services. Thus, the review question was: what are the main relevant aspects and results of applying AI in healthcare for vulnerable populations who are victims of interpersonal violence?

Two reviewers performed independent search and extraction. Cases of discrepancies (two occurrences) were resolved by a third researcher. The following descriptors from Medical Subject Headings were used: “Artificial Intelligence”; “Interpersonal Violence”; “Vulnerable Population”; and “Marginalized Population”. The following descriptors in Portuguese from the Health Sciences Descriptors were used: “*Violência*”; “*Violência Interpessoal*”; “*Violência Contra Mulher*”; “*Vulnerabilidade Social*”; and “*Inteligência Artificial*”.

For searches in other databases, modifications were made according to their specificities. The descriptors were combined in various ways to broaden the searches, using terminological variations and synonyms in the listed languages. The combination of descriptors was performed using the Boolean operators AND (restrictive combination) and OR (additive combination). For keywords with the same acronym as the PCC strategy, OR was used, and for combinations between different acronyms, AND was used, as shown in Chart 1.

**Chart 1:** Search strategy in databases, São Paulo, São Paulo, Brazil, 2025

PubMed^*^	(“artificial intelligence”[MeSH Terms] OR (“artificial”[All Fields] AND “intelligence”[All Fields]) OR “artificial intelligence”[All Fields]) AND (“violence”[MeSH Terms] OR “violence”[All Fields] OR (“interpersonal”[All Fields] AND “violence”[All Fields]) OR “interpersonal violence”[All Fields]) AND (“vulnerable populations”[MeSH Terms] OR (“vulnerable”[All Fields] AND “populations”[All Fields]) OR “vulnerable populations”[All Fields] OR (“vulnerable”[All Fields] AND “population”[All Fields]) OR “vulnerable population”[All Fields]) AND (“marginality”[All Fields] OR “marginalization”[All Fields] OR “marginalizations”[All Fields] OR “marginalize”[All Fields] OR “marginalized”[All Fields] OR “marginalizes”[All Fields] OR “marginalizing”[All Fields]) AND (“populate”[All Fields] OR “populated”[All Fields] OR “populates”[All Fields] OR “populating”[All Fields] OR “population”[MeSH Terms] OR “population”[All Fields] OR “population groups”[MeSH Terms] OR (“population”[All Fields] AND “groups”[All Fields]) OR “population groups”[All Fields] OR “populations”[All Fields] OR “population s”[All Fields] OR “populational”[All Fields] OR “populations s”[All Fields] OR “populous”[All Fields])
WoS^**^, Embase^***^, PsycINFO - APA PsycNET^#^	artificial AND intelligence AND (‘intelligence’/exp OR intelligence) AND interpersonal AND (‘violence’/exp OR violence) AND vulnerable AND marginalized AND (‘population’/exp OR population)
**CINAHL^##^**	artificial intelligence AND interpersonal violence AND vulnerable population OR marginalized population

* National Library of Medicine;

** Web of Science;

*** Excerpta Medica DataBASE;

# American Psychological Association;

## Cumulative Index to Nursing and Allied Health Literature.

### Analysis of results

For data collection, a validated and adapted instrument was used, whose variables included title, authors, year of publication and journal, language, objectives, design, and main results^(13)^. The variables mapped in relation to AI were model type, implementation environment, training data, and usability. The methodological quality analysis and risk of bias of selected studies were performed using JBI Appraisal Tools^(14)^. Methodological quality assessment allowed for a quick visualization and assessment of the study’s robustness. Articles with 80% to 100% correspondence to JBI Appraisal Tools criteria indicated high methodological quality; between 60% and 79% indicated moderate quality; and below 59% indicated low quality. The results were analyzed descriptively, with a synthesis of included studies.

## RESULTS

**Figure 1:**
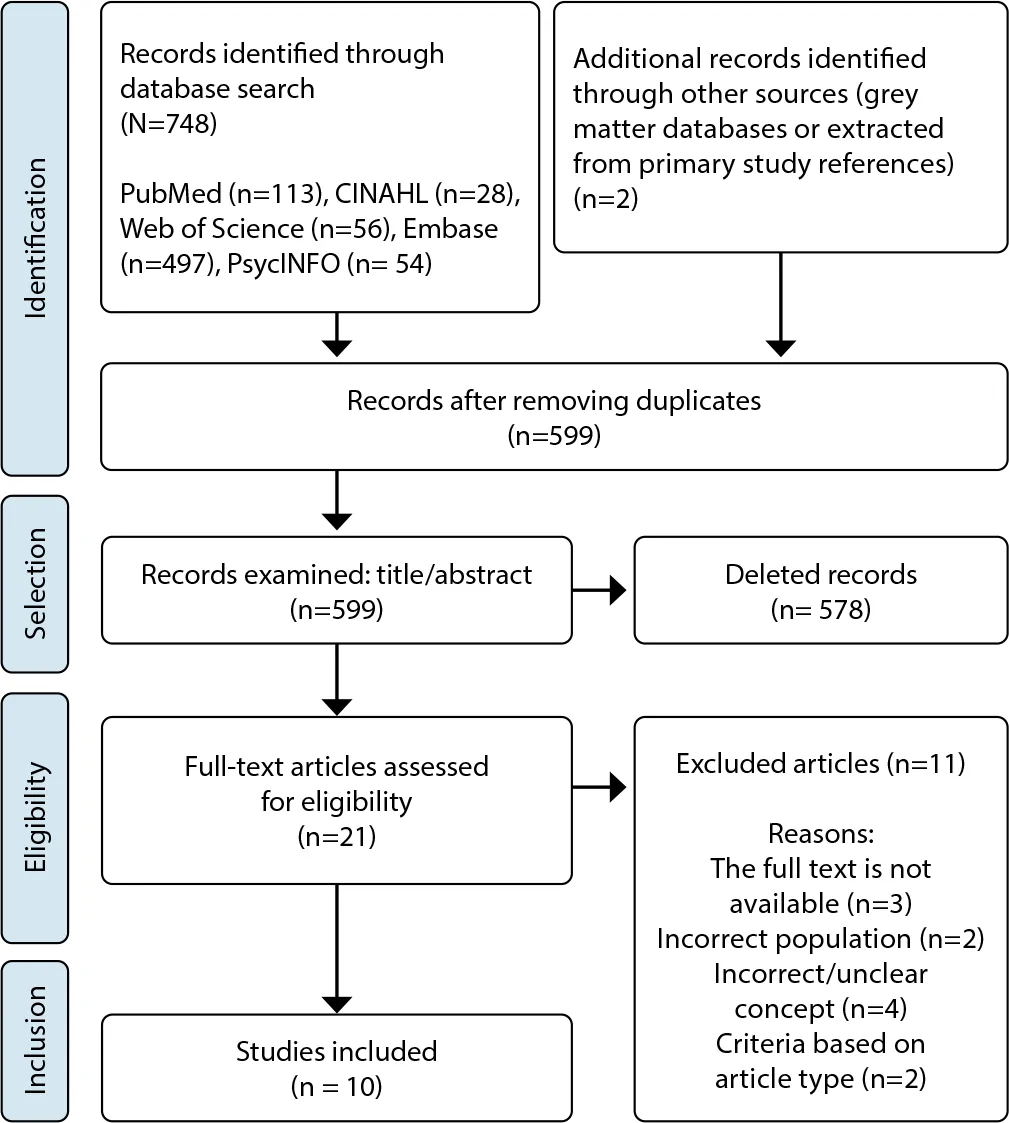
Flowchart based on the Preferred Reporting Items for Systematic Reviews and Meta-Analyses Extension for Scoping Reviews criteria according to JBI, Brazil, 2024

The selection process began with the identification of 748 records in databases, in addition to two additional records from grey literature or references. After removing duplicates, 599 records remained for the selection phase, of which 578 were excluded after title and abstract analysis. In the eligibility phase, 21 full-text articles were assessed, resulting in the exclusion of 11 studies due to reasons such as unavailability of text, incorrect population, inadequate concept, or article type criteria. In the end, ten studies were included in the analysis. The flowchart in Figure 1 summarizes the article selection process.

All studies included in the review (n=10) were published in English. Concerning geographical distribution, the articles encompass researchers from various countries, including Spain, Canada, the United States of America, Liberia, Norway, Turkey, South Africa, Mexico, Colombia, and India, reflecting a global interest in the application of AI to address violence. Research methods varied between theoretical essays (n=2; 20%), reviews (n=2; 20%), methodological studies (n=1; 10%), mixed methods (n=1; 10%), document analysis (n=1; 10%), cross-sectional study (n=1; 10%), assessment study (n=1; 10%), and experience report (n=1; 10%). Publications occurred between 2014 and 2025, with a concentration in 2024 (n=4; 40%). Chart 2 presents detailed summaries of selected studies.

**Chart 2:** Synthesis of selected materials, São Paulo, São Paulo, Brazil, 2025

Year	Authors/title	Objective	Study design	Outcomes	Methodological quality
2023	Garcia-Vergara E, Almeda N, Fernández-Navarro F, Becerra-Alonso D^(15)^ Spain Artificial intelligence extracts key insights from legal documents to predict intimate partner femicide	Identify relevant information in legal documents to predict femicide by an intimate partner using artificial intelligence.	Document analysis	Machine learning and natural language processing models showed a detection rate of over 75% for both lethal and non-lethal (high-severity) cases, proving useful for predicting victim risk.	High
2025	Abdulai AF^(16)^ Canada Is Generative AI Increasing the Risk for Technology-Mediated Trauma Among Vulnerable Populations?	Examine how digital health interventions powered by generative artificial intelligence can generate emotional trauma among vulnerable populations and to present strategies to mitigate such trauma in the context of digital health interventions.	Theoretical essay	Content generated by artificial intelligence, such as health advice or automated interactions, can inadvertently trigger or reactivate traumatic experiences in vulnerable individuals, such as victims of violence. Artificial intelligence developers and researchers should adopt trauma-informed approaches and consider the specific needs of vulnerable populations and victims of violence when designing and implementing artificial intelligence technologies in healthcare.	Moderate
2024	Thomas A, Asnes A, Libby K, Hsiao A, Tiyyagura G^(17)^ United States of America Developing and Testing the Usability of a Novel Child Abuse Clinical Decision Support System: Mixed Methods Study	Develop and test the usability of a clinical decision support system for child abuse cases based on natural language processing, known as Child Abuse Clinical Decision Support.	Mixed method	The system’s user-centered design and evidence-based content have helped professionals recognize and assess child physical abuse in real time, and have the potential to reduce the number of missed cases.	High
2024	Rahman R *et al*.^(18)^ Liberia A comparative study of machine learning algorithms for predicting domestic violence vulnerability in Liberian women	Predicting women’s vulnerability to domestic violence using a machine learning approach and data from the Liberia Demographic and Health Survey.	Assessment study	The Light Gradient-Boosting Machine and Random Forest were the artificial intelligence models that achieved the highest accuracy in predicting women’s vulnerability to domestic violence, with accuracy rates of 81% and 82%, respectively, especially regarding emotional violence.	High
2014	Hammer HL^(19)^ Norway Detecting Threats of Violence in Online Discussions Using Bigrams of Important Words	Develop an automated method to identify threats of violence in online discussions using machine learning.	Experience report	24.840 sentences from YouTube comments were collected and manually annotated as containing or not containing violent threats. This data was used to train and test machine learning models. The trained model showed an error rate of approximately 10% when classifying a violent sentence as non-violent, and a 5% error rate when classifying non-violent sentences as violent. The inclusion of bigrams composed of important words and consideration of the distance between these words in the sentence resulted in the best classification performance.	Moderate
2024	Başaran F, Duru P^(20)^ Turkey Determining domestic violence against women using machine learning methods: The case of Türkiye	Determine the frequency and risk factors of domestic violence against women using machine learning methods.	Cross-sectional	The performance of machine learning algorithms showed sensitivity between 91% and 100% and specificity between 85% and 100% in the 630 cases analyzed. Length of marriage (after ten years of union), a high number of children, and a low level of education were suggested as indicators of risk of violence by the artificial intelligence.	High
2020	Hunt X *et al.* ^(21)^ South Africa Artificial Intelligence, Big Data, and mHealth: The Frontiers of the Prevention of Violence Against Children	Map the main scientific literature on the use of artificial intelligence for the prevention of violence against children.	Review	Machine learning combined with big data is seen as having promising potential for reducing violence against children, especially in lowand middle-income countries. The models should encompass information from large territories (entire cities) on predictors of violence to generate prevention and control algorithms. Ethical aspects, such as confidentiality and access control, are also highlighted for the implementation of artificial intelligence systems.	Moderate
2023	Cruz-Mendoza MC *et al*.^(22)^ Mexico Machine Learning Applied to Improve Prevention of, Response to, and Understanding of Violence Against Women	Describe a web application based on machine learning to collect cases of intimate partner violence and predict severity.	Methodological	The Random Forest model stood out as the best tool for identifying cases of domestic violence, achieving a 97% accuracy rate. The application diagnosed the cases and generated maps highlighting the areas with the highest risk of violence.	Moderate
2025	Pinto-Muñoz CC *et al*.^(23)^ Colombia Machine Learning Applied to Gender Violence: A Systematic Mapping Study	Identify methodological trends and systematically map the use of machine learning applied to gender-based violence.	Review	Natural language processing and predictive models are the main methodological trends. The results indicated that artificial intelligence models have a high predictive capacity, representing a significant improvement compared to risk assessment systems currently in use. There is evidence of few studies on the subject in Latin America and the Caribbean.	High
2024	Tiwari A K, Marisport A^(24)^ India Leveraging Artificial Intelligence to Address Domestic Violence Against Women with Disabilities in India	Identify how artificial intelligence technology can enhance detection tactics and prevent domestic violence against women with disabilities in India.	Theoretical essay	Mobile applications for reporting abuse and virtual assistants for providing information and support can offer predictive analytics to assist health and safety authorities in decision-making. The security of victims’ information is fundamental to preventing data leaks that increase their vulnerability.	Moderate

Study methodological assessment demonstrated that most presented high or moderate quality, with varied designs. This diversity made direct comparison of the samples impossible, but contributed to a comprehensive overview of the several ways in which AI can be applied to combat violence.

The populations most frequently addressed were women experiencing domestic violence, children who were victims of abuse, and people with disabilities. Some studies also addressed populations in socioeconomic vulnerability or residents of lowand middle-income countries, where technological inequalities have made the use of AI even more relevant.

From the analysis of the findings, three major topics emerged: 1) Prediction and automated monitoring of violence^(15,18,20,22,23)^; 2) Decision support, technological innovation in health and social protection^(17,21,24)^; and 3) Ethics, security, and social impact of artificial intelligence in vulnerable contexts^(16,19)^.

Studies in the “Prediction and automated monitoring of violence” category mention that machine learning and natural language processing (NLP) models have achieved high accuracy in predicting and identifying cases of interpersonal violence, with success rates between 75% and 97%^(15,18,20,22,23)^. The tools developed were able to recognize risk patterns from legal, demographic, and behavioral data, highlighting factors such as prolonged marriage, low educational level, and a high number of children.

The studies explored the use of bigrams and text mining techniques in judicial records and social networks, which allowed for the identification of threats of violence and the generation of real-time risk maps, demonstrating AI’s ability to recognize linguistic and behavioral patterns associated with interpersonal violence. This reinforces AI’s potential as a surveillance tool capable of anticipating dangerous situations and supporting public security and health policies^(15,18,20,22,23)^.

The effectiveness of these models depended on large, contextualized, and culturally sensitive databases, as well as requiring constant validation to avoid biases and misinterpretations^(18,20,23)^. AI has not emerged as a replacement for human analysis, but as an ally in recognizing complex patterns and preventing violent events^(15,18,20,22,23)^.

In the “Decision support, technological innovation in health and social protection” category, studies highlighted the use of AI as a tool to support care and decision-making in contexts of violence. The development of mobile applications and virtual assistants has enabled reporting abuse, seeking information, and receiving guidance in a safe and accessible manner^(21,24)^. In countries with limited infrastructure, the combination of machine learning, big data, and mobile health technologies has shown potential to reduce underreporting of cases and strengthen social safety nets.

These initiatives brought technology closer to healthcare practice, making AI a tool for humanized care. Studies, however, emphasized that the advancement of these solutions required investments in technological infrastructure, professional training, and data governance policies that ensured confidentiality and equitable access to the tools.

The studies grouped in the “Ethics, security, and social impact of artificial intelligence in vulnerable contexts” category brought ethical and emotional reflections on the use of AI in matters of violence. It was observed that automated interactions, such as AI-generated advice, could reactivate traumatic experiences in victims and survivors if not carefully designed. Information leaks and misuse of records generated new forms of vulnerability, transforming what should be a protective tool into a source of risk. The creation of robust ethical protocols, algorithmic transparency, and social participation in the development and implementation stages were reported as essential^(16,19)^.

These aspects reinforced the fact that behind each piece of data analyzed, there were real people with unique stories and struggles, and that technology only fulfilled its role when it managed to protect and respect those lives.

## DISCUSSION

Although research has been concentrated in the United States, the presence of research in various regions of the world, concentrated in recent years, shows that the topic is of global interest and quite contemporary, and the debate revolves around the potential and also the limitations of intelligent systems.

The findings of this review engage critically and consistently with a growing body of international scientific literature. The accuracy (75%-97%) of automated prediction and monitoring models supports international studies that highlight machine learning’s and NLP’s potential to identify complex risk patterns. Research conducted in the United States used text mining on social service records, demonstrating how algorithms can predict the recurrence of child maltreatment with significant accuracy, supporting case screening^(25)^. However, the caveat in the results regarding the reliance on “broad, contextualized and culturally sensitive” databases^(18,20,23)^ refers to the warnings described in the literature. A North American study shows how predictive models in health have erroneously prioritized white patients over black patients with equivalent clinical needs^(26)^, a bias that can be replicated in violence screening systems, further underreporting marginalized groups.

The promise of effective predictive surveillance also faces challenges in practical implementation. While results point to the generation of “real-time risk maps”^(15,19,22,23)^, Australian researchers warn that the expansion of mobile health technologies, without a privacy protection framework, may expose victims to new risks, such as unauthorized access to their data by abusers, a situation that is particularly critical in the context of domestic violence^(27)^. This vulnerability transforms protective devices into instruments of risk, requiring that technological development go hand in hand with digital security.

Researchers in the United Kingdom also warn of the risk of “selective hyper-surveillance” in already marginalized communities, where the presence of security forces is historically greater and trust is much lower^(28)^. This practice can criminalize poverty and perpetuate cycles of traumatic intervention, instead of channeling resources toward social and community support. Therefore, AI’s technical effectiveness in predicting violence must be constantly balanced with a critical analysis of its social impact and its potential to reinforce stigmas.

The results indicated that AI is an “ally”, not a replacement, for human analysis^(15,18,20,22,23)^. This factor is important because studies conducted in Europe with chatbots supporting victims of gender-based violence have found that, while useful for basic information, these tools often fail to adequately respond to complex scenarios involving psychological violence or intersectionality, disregarding contextual nuances that are fundamental for providing humane support^(29)^.

The ethical and emotional reflections grouped in the third category of results^(16,19)^ constitute perhaps the point of greatest convergence with the most forceful criticisms. The finding that automated interactions can reactivate traumas is in line with studies that question AI systems’ ability to interpret subtexts and cultural nuances. The emphasis on the need for “robust ethical protocols and algorithmic transparency”^(16,19)^ is a direct consequence of the debate on “black box systems”. Canadian researchers mention that overcoming these challenges requires a participatory and multi-stakeholder governance approach, incorporating perspectives of victims, civil society, and ethics experts^(30)^. These researchers argue that algorithmic impact assessment should be a mandatory requirement before deploying any system in vulnerable contexts^(30)^.

The findings regarding the need for protocols and governance find synergy in comparative legal analyses. While the European Union is advancing with its Artificial Intelligence Act, which classifies high-risk systems and imposes transparency requirements, and Brazil has the General Data Protection Law, experts argue that neither of these frameworks is specific enough to address the unique risks faced by victims of violence assisted by AI^(31)^. The development of sector-specific regulations, which provide for independent audits, dispute mechanisms, and the prohibition of particularly harmful uses, therefore becomes a global urgency.

Thus, the findings of this study reflect the state of the art in the application of AI, as well as mirroring the main axes of tension identified by the global scientific literature. The way forward, therefore, does not lie in simple technological adoption, but in its critical and regulated integration. AI does not present itself as a panacea, but as a tool that, to fulfill its promise of protection, must be continuously assessed, regulated, and subjected to democratic assessment, ensuring that algorithmic efficiency never overrides the guarantee of human rights and dignity of victims.

### Study limitations

This study has some important limitations. Although the main health databases were consulted and grey matter searches were conducted, articles on the subject may be indexed in other databases that were not accessed. Another limitation was the wide range of methods used in the research that comprised the sample, making it impossible to assess successful measures, both in absolute and relative numbers. It is noteworthy that the limited mention in the selected studies of some other vulnerable populations, such as black people, the LGBTQIA+ community, and people experiencing homelessness, among others, makes it difficult to infer about appropriate measures for these populations.

### Contributions to nursing and science

Despite the limitations mentioned, the study proves to be highly relevant and current from the perspective that the topic of AI is proving to be an additional tool in addressing the problem, important worldwide and with a high impact on the most vulnerable populations.

## CONCLUSIONS

This review aimed to map the main applications of AI in combating interpersonal violence against vulnerable populations. It was found that machine learning and NLP systems achieved significant accuracy in prediction and monitoring, although their effectiveness depended directly on representative and culturally sensitive databases, at the risk of amplifying social biases. The risk of “selective hyper-surveillance” in already marginalized communities could criminalize poverty, divert attention from the objective of addressing the issue, and increase prejudice.

Findings regarding ethical aspects indicated that automated interactions could reactivate traumas, highlighting the risk to privacy, as the lack of protection in mobile technologies can expose victims to new dangers, such as unauthorized access to their data by aggressors. Overcoming these challenges requires participatory and multi-sectoral governance whenever possible. The need for ethical protocols and transparency was pointed out, as well as the assertion that existing regulatory frameworks are not sufficiently specific. Legal regulations for the use of AI are necessary, according to the reality of each country, but always respecting the principles of justice, beneficence, and respect for autonomy. Epidemiological and interventional studies with better levels of evidence are fundamental for advancing the topic, with an increase in the inclusion of other highly vulnerable populations, such as black people, members of the LGBTQIA+ community, people experiencing homelessness, and other minorities.

## Data Availability

The research data are available only upon request.

## References

[1] Rubenstein BL, Lu LZN, MacFarlane M, Stark L. (2020). Predictors of interpersonal violence in the household in humanitarian settings: a systematic review. Trauma Violence Abuse.

[2] Souza NR, Hino P, Taminato M, Okuno MFP, Gogovor A, Fernandes H. (2024). Violence against brown and black women during the pandemic: a scoping review. Acta Paul Enferm.

[3] Palmer JE, Williams E, Mennicke A. (2022). Interpersonal violence experiences and disclosure patterns for lesbian, gay, bisexual, queer+, and heterosexual university students. J Family Violence.

[4] Pan American Health Organization (PAHO) (2025). Global Database on the Prevalence of Violence Against Women.

[5] Ranzani CM, Silva SC, Hino P, Tamianto M, Okuno MFP, Fernandes H. (2023). Profile and characteristics of violence against older adults during the COVID-19 pandemic. Rev Latino-Am Enfermagem.

[6] Stubbs A, Szoeke C. (2022). The effect of intimate partner violence on the physical health and health-related behaviors of women: a systematic review of the literature. Trauma, Violence Abuse.

[7] Notko M, Husso M, Piippo S, Fagerlund M, Houtsonen J. (2021). Intervening in domestic violence: interprofessional collaboration among social and health care professionals and the police. J Interprof Care.

[8] Youngson N, Saxton M, Jaffe PG, Chiodo D, Dawson M, Straatman AL. (2021). Challenges in risk assessment with rural domestic violence victims: implications for practice. J Fam Viol.

[9] Hunt X, Tomlinson M, Sikander S, Skeen S, Marlow M, Toit S, Eisner M. (2020). Artificial Intelligence, Big Data, and mHealth: the frontiers of the prevention of violence against children. Front Artif Intell.

[10] Minhas R, Elphick C, Shaw J. (2022). Protecting victim and witness statement: examining the efectiveness of a chatbot that uses artifcial intelligence and a cognitive interview. AI Soc.

[11] Santos WM, Secoli SR, Püschel VAA. (2018). The Joanna Briggs Institute approach for systematic reviews. Rev Latino-Am Enfermagem.

[12] Tricco AC, Lillie E, Zarin W, O’Brien KK, Colquhoun H, Levac D (2018). PRISMA Extension for Scoping Reviews (PRISMA-ScR): checklist and explanation. Ann Intern Med.

[13] Ursi ES, Gavão CM. (2006). Perioperative prevention of skin injury: an integrative literature review. Rev Latino-Am Enfermagem.

[14] Joanna Briggs Institute (JBI) (2022). Critical Appraisal Tools.

[15] Garcia-Vergara E, Almeda N, Fernández-Navarro F, Becerra-Alonso D. (2024). Artificial intelligence extracts key insights from legal documents to predict intimate partner femicide. Artif Intell Rev.

[16] Abdulai AF. (2025). Is Generative AI Increasing the Risk for Technology-Mediated Trauma Among Vulnerable Populations?. Nurs Inq.

[17] Thomas A, Asnes A, Libby K, Hsiao A, Tiyyagura G. (2024). Developing and testing the usability of a novel child abuse clinical decision support system: mixed methods study. J Med Internet Res.

[18] Rahman M, Hossain MS, Uddin S, Hossain MA, Jameel H. (2024). A comparative study of machine learning algorithms for predicting domestic violence vulnerability in Liberian women. BMC Womens Health.

[19] Rodriguez JA, Alsentzer E, Bates DW. (2024). Leveraging large language models to foster equity in healthcare. J Am Med Inform Assoc.

[20] Başaran F, Duru P. (2024). Determining domestic violence against women using machine learning methods: the case of Turkye. Arch Psychiatr Nurs.

[21] Tilmon S, Nyenhuis S, Solomonides A, Barbarioli B, Bhargava A, Birz S (2023). Sociome Data Commons: a scalable and sustainable platform for investigating the full social context and determinants of health. J Clin Transl Sci.

[22] Cruz-Mendoza MC, Melendez-Armenta RA, Canul-Reich J, Muñoz-Benítez J. (2025). Machine Learning Applied to Improve Prevention of, Response to, and Understanding of Violence Against Women. Informatics.

[23] Pinto-Muñoz CC, Benito-Santos A, Vidal JC. (2024). Machine learning applied to gender violence: a systematic mapping study. Heliyon.

[24] Chouldechova A, Benavides-Prado D, Fialko O, Vaithianathan R. (2018). A case study of algorithm-assisted decision making in child maltreatment hotline screening decisions. Proceedings of the 1st Conference on Fairness, Accountability and Transparency. In: Proceedings of Machine Learning Research.

[25] Obermeyer Z, Powers B, Vogeli C, Mullainathan S. (2019). Dissecting racial bias in an algorithm used to manage the health of populations. Science.

[26] Freed D, Palmer J, Minchala D, Levy K, Ristenpart T, Dell N. (2018). “A Stalker’s Paradise”: how intimate partner abusers exploit technology. In: Proceedings of the 2018 CHI Conference on Human Factors in Computing Systems.

[27] Ferguson AG. (2017). The Rise of Big Data Policing: Surveillance, Race, and the Future of Law Enforcement. New York University Press;.

[28] Mielismäki H, Husso M. (2025). Ethical implications of AI-driven chatbots in domestic violence support. Soc Inclus.

[29] Metcalf J, Moss E. (2019). Owning Ethics: Corporate Logics, Silicon Valley, and the Institutionalization of Ethics. Soc Res (New York).

[30] Veale M, Zuiderveen Borgesius F. (2021). Demystifying the Draft EU Artificial Intelligence Act. Comput Law Security Ver.

