# Supplementary material for: Artificial Intelligence in Addressing Interpersonal Violence Against Vulnerable Populations: A Scoping Review
Source: Rev Bras Enferm. 2026 Aug 21;79:e20250547. doi: 10.1590/0034-7167-2025-0547 (PMC13505558; doi:10.1590/0034-7167-2025-0547)
Supplement: Translation

# Translation: Inteligência Artificial no Enfrentamento da Violência Interpessoal Contra Populações Vulneráveis: Revisão de Escopo

**DOI:** 10.1590/0034-7167-2025-0547pt

**Authors:** Hallessa de Fátima da Silva Pimentel, Julia Sunmy Aguni Chunga, Ana Maria José Garcia João Pascoal, Dulce Aparecida Barbosa, Claudia Galindo Novoa, Hugo Fernandes

**Affiliations:** I Universidade Federal de São Paulo. São Paulo, São Paulo, Brasil; II Universidade Agostinho Neto. Talatona, Angola

**Keywords:** Inteligência Artificial, Violência, Populações Vulneráveis, Sistemas Computacionais, Saúde Pública.

## Abstract

**Objetivos:**

mapear e sumarizar as principais aplicações da inteligência artificial no enfrentamento da violência interpessoal contra populações vulneráveis.

**Métodos:**

revisão de escopo, realizada entre dezembro de 2024 e fevereiro de 2025 nas bases de dados PubMed, *Web of Science*, Embase, PsycINFO e CINAHL, além de bases de materiais cinzentos. Excluíram-se editoriais, retratações e estudos de violência autoprovocada.

**Resultados:**

750 textos foram encontrados. Após aplicação dos critérios de elegibilidade, foi obtida uma amostra de dez materiais. Os estudos foram agrupados nas categorias “Predição e monitoramento automatizado da violência”, “Apoio à decisão, inovação tecnológica em saúde e proteção social” e “Ética, segurança e impacto social da inteligência artificial em contextos de vulnerabilidade”.

**Conclusões:**

modelos de inteligência artificial sobre enfrentamento da violência contra populações vulneráveis exigem verificação, proteção de dados, governança social e testes com alto nível de evidência.

## INTRODUÇÃO

A violência interpessoal é qualquer ação intencional que cause danos a outra pessoa, sejam eles físicos, psicológicos, sexuais, morais ou patrimoniais. Essa violência pode ocorrer em diversos contextos, como família, escola, trabalho e comunidade, e envolve o uso da força, poder, ameaças ou outras formas de coerção para controlar, dominar ou prejudicar alguém. Trata-se de um agravo à saúde complexo com diversas causas e consequências, e a desigualdade social, machismo, racismo e outros tipos de discriminação podem contribuir para o seu aumento^(1)^.

Pode existir um ciclo de retroalimentação que envolve a violência e vulnerabilidade social. Indivíduos que vivenciam condições de pobreza, desigualdade social, discriminação ou exclusão são significativamente mais suscetíveis a serem vítimas de violência. A classe social e econômica, por exemplo, pode limitar o acesso a recursos básicos como educação, saúde e segurança, tornando as pessoas mais vulneráveis a diversas formas de violência^(2)^. Além disso, a desigualdade social cria um ambiente propício à discriminação e exclusão, marginalizando grupos específicos como mulheres, negros, idosos, crianças, pessoas da comunidade de lésbicas, *gays*, bissexuais, transexuais e transgêneros, *queers*, pessoas intersexo, assexuais e demais minorias sexuais (LGBTQIA+), além de pessoas em situação de rua. Essa marginalização os torna alvos fáceis de agressores, que encontram na vulnerabilidade dessas pessoas uma oportunidade para exercer poder e dominação^(2,3)^. A interseccionalidade da vulnerabilidade social, ou seja, a combinação de diferentes marcadores sociais como raça, gênero, classe e orientação sexual, agrava ainda mais essa situação, expondo essas pessoas a múltiplas formas de opressão e violência.

A Organização Panamericana de Saúde estima que, só no caso das mulheres, uma em cada três mulheres entre 15 e 49 anos já sofreu violência física e/ou sexual, e no caso de menores de 15 anos, uma em cada quatro delas já sofreu a mesma injúria, sendo os familiares ou conhecidos os principais agressores^(4)^. Pesquisadores brasileiros afirmam que as pessoas idosas também têm elevada incidência de violência interpessoal perpetrada por familiares, mas que as mais comuns incluem a tipologia psicológica ou moral e financeira^(5)^. Já em relação à comunidade LGBTQIA+, estudiosos apontam que transgêneros têm seus direitos humanos gravemente lesados, sendo constantemente alvos de agressões físicas, morais e sexuais^(3)^.

Pesquisadores australianos citam que os profissionais de saúde ainda possuem muita dificuldade na tomada de decisão rápida e assertiva no atendimento a vítimas de violência, especialmente àquelas que possuem muitos determinantes sociais envolvidos^(6)^. Mesmo quando há fluxos claros e bem estabelecidos, nem sempre os profissionais de saúde que estão responsáveis pelo primeiro atendimento sabem quais as condutas prioritárias e quais encaminhamentos devem ser realizados para redução de danos às vítimas e testemunhas. As justificativas podem ser desde pouco treinamento sobre as linhas de cuidado e/ou pouca empatia até dificuldades de priorizar para quais locais destinar essas pessoas, tais como às delegacias especializadas, serviços de proteção, entre outros^(7,8)^.

Uma tecnologia mais recente que parece ser promissora para redução de erros e melhoria no atendimento a vítimas em serviços de saúde, especialmente em casos complexos ou nos quais a decisão do profissional de saúde precisa ser muito rápida, é a inteligência artificial (IA). A utilização de redes neurais, treinamento de máquinas e modelos de linguagem tem sido experimentada por pesquisadores como uma ferramenta auxiliar no enfrentamento dessa realidade^(9,10)^. Porém, por se tratar de um recurso relativamente novo na área da saúde, sua implementação pode envolver cuidados especiais, como o armazenamento sigiloso dos dados de um caso de violência.

Em buscas nas plataformas *Open Science Framework* e *International Prospective Register of Systematic Reviews*, não foram obtidos registros de revisões sistemáticas e/ou de escopo sobre a temática, podendo sinalizar um potencial ineditismo que motiva o desenvolvimento desta investigação. Assim, a relevância desta pesquisa está em compilar as evidências sobre o enfrentamento da violência interpessoal contra populações de maior vulnerabilidade social, mediadas pela IA.

## OBJETIVOS

Mapear e sumarizar as principais aplicações da IA no enfrentamento da violência interpessoal contra populações vulneráveis.

## MÉTODOS

### Aspectos éticos da pesquisa

As legislações nacionais e internacionais sobre ética em pesquisas com seres humanos foram integralmente respeitadas. Devido à natureza da pesquisa, que não envolveu contato direto com pessoas ou animais, dispensaram-se a submissão e aprovação por um Comitê de Ética em Pesquisa. Contudo, realizou-se o preenchimento de uma declaração de responsabilidade e avaliação pelo Departamento de Saúde Pública, em consonância com a Resolução n^o^ 200 de 2021 do Conselho Universitário da Universidade Federal de São Paulo.

### Desenho do estudo e período

Trata-se de revisão de escopo da literatura, estruturada de acordo com as recomendações do JBI^(11)^, que foram: 1) pesquisa exploratória inicial para encontrar referências primárias, que são registros de estudos que atenderam aos critérios de inclusão para a questão da revisão; 2) seguimento de uma estratégia de pesquisa abrangente usando descritores indexados principais do protocolo; e 3) busca suplementar de literatura cinzenta, que incluiu pesquisa de citações e pesquisa manual.

A coleta de dados foi realizada entre dezembro de 2024 e fevereiro de 2025. Foram feitas buscas nas bases de dados *National Library of Medicine* (PubMed), *Web of Science, Excerpta Medica DataBASE* (Embase), PsycINFO - APA PsycNET (*American Psychological Association*) e *Cumulative Index to Nursing and Allied Health Literature* (CINAHL). Foram pesquisadas referências cinzentas em teses e dissertações de acesso público e irrestrito no *MedNar, WorldWideScience* e *Google Scholar*. Também foram incluídos estudos adicionais a partir de referências dos artigos primários (pesquisa manual).

### Critérios de inclusão e exclusão

Incluíram-se estudos sobre IA e violência interpessoal contra populações vulneráveis (mulheres, crianças, idosos, pessoas em situação de rua, população negra e membros da comunidade LGBTQIA+), publicados nos idiomas português, espanhol ou inglês, sem recorte temporal. Foram excluídos editoriais, retratações e estudos voltados à violência autoprovocada ou tentativas de suicídio. O nível de evidência não foi considerado critério de exclusão, justificando se tratar de um tema ainda recente na área da saúde. O relatório de revisão foi elaborado de acordo com o *checklist Preferred Reporting Items for Systematic Reviews and Meta-Analyses Extension for Scoping Reviews: Checklist and Explanation* ^(12)^.

### Protocolo do estudo

Para elaboração da questão de revisão, foram seguidas as seguintes fases: identificação da questão; busca por estudos relevantes; seleção e extração dos dados; e agrupamento, resumo e apresentação dos resultados. Adotou-se a estratégia PCC (*Population, Concept and Context*) para formulação da pergunta da pesquisa com as seguintes representações: P = populações vulneráveis (mulheres, crianças, idosos, pessoas em situação de rua, população negra e membros da comunidade LGBTQIA+) vítimas de violência; C = IA; e C = serviços de saúde. Dessa forma, a questão de revisão foi: quais são os principais aspectos relevantes e resultados da aplicação da IA no atendimento à saúde populações vulneráveis vítimas de violência interpessoal?

Dois revisores realizaram busca e extração independentes. Casos de divergências (duas ocorrências) foram resolvidos por um terceiro pesquisador. Utilizaram-se os seguintes descritores do *Medical Subject Headings:* “*Artificial Intelligence*”; *Interpersonal Violence*”; “*Vulnerable Population*”; e “*Marginalized Population*”. Utilizaram-se os seguintes descritores do Descritores em Ciências da Saúde: “Violência”; “Violência Interpessoal”; “Violência Contra Mulher”; “Vulnerabilidade Social”; e “Inteligência Artificial”.

Para buscas nas outras bases de dados, foram feitas modificações de acordo com suas especificidades. Os descritores foram combinados de formas variadas para ampliar as buscas, usando variações terminológicas e sinônimos nos idiomas elencados. A combinação dos descritores foi realizada com uso dos operadores booleanos *AND* (combinação restritiva) e *OR* (combinação aditiva). Entre as palavras-chave do mesmo acrônimo da estratégia PCC, foi usado o *OR*, e para a combinação entre acrônimos diferentes, o *AND*, conforme Quadro 1.

**Quadro 1:** Estratégia de busca nas bases de dados, São Paulo, São Paulo, Brasil, 2025

PubMed^*^	*(“artificial intelligence”[MeSH Terms] OR (“artificial”[All Fields] AND “intelligence”[All Fields]) OR “artificial intelligence”[All Fields]) AND (“violence”[MeSH Terms] OR “violence”[All Fields] OR (“interpersonal”[All Fields] AND “violence”[All Fields]) OR “interpersonal violence”[All Fields]) AND (“vulnerable populations”[MeSH Terms] OR (“vulnerable”[All Fields] AND “populations”[All Fields]) OR “vulnerable populations”[All Fields] OR (“vulnerable”[All Fields] AND “population”[All Fields]) OR “vulnerable population”[All Fields]) AND (“marginality”[All Fields] OR “marginalization”[All Fields] OR “marginalizations”[All Fields] OR “marginalize”[All Fields] OR “marginalized”[All Fields] OR “marginalizes”[All Fields] OR “marginalizing”[All Fields]) AND (“populate”[All Fields] OR “populated”[All Fields] OR “populates”[All Fields] OR “populating”[All Fields] OR “population”[MeSH Terms] OR “population”[All Fields] OR “population groups”[MeSH Terms] OR (“population”[All Fields] AND “groups”[All Fields]) OR “population groups”[All Fields] OR “populations”[All Fields] OR “population s”[All Fields] OR “populational”[All Fields] OR “populations s”[All Fields] OR “populous”[All Fields])*
WoS^**^, Embase^***^, PsycINFO - APA PsycNET^#^	*artificial AND intelligence AND (‘intelligence’/exp OR intelligence) AND interpersonal AND (‘violence’/exp OR violence) AND vulnerable AND marginalized AND (‘population’/exp OR population)*
**CINAHL^##^**	*artificial intelligence AND interpersonal violence AND vulnerable population OR marginalized population*

* National Library of Medicine;

** Web of Science;

*** Excerpta Medica DataBASE;

# American Psychological Association;

## Cumulative Index to Nursing and Allied Health Literature.

### Análise dos resultados

Para a coleta de dados, foi utilizado instrumento validado adaptado, cujas variáveis incluíram título, autores, ano de publicação e periódico, idioma, objetivos, delineamento, e principais resultados^(13)^. As variáveis mapeadas em relação à IA foram tipo de modelo, ambiente de implementação, dados de treino e usabilidade. A análise da qualidade metodológica e o risco de viés dos estudos selecionados foram feitos com auxílio das ferramentas JBI *Appraisal Tools* ^(14)^. A avaliação da qualidade metodológica permitiu visualizar rapidamente e avaliar a robustez do estudo. Artigos com correspondência de 80% a 100% aos critérios do JBI *Appraisal Tools* indicaram alta qualidade metodológica; entre 60% e 79%, indicaram qualidade moderada; e abaixo de 59%, indicaram baixa qualidade. Os resultados foram analisados descritivamente, com a síntese dos estudos incluídos.

## RESULTADOS

O processo de seleção iniciou-se com a identificação de 748 registros em bases de dados, além de dois registros adicionais provenientes de literatura cinzenta ou referências. Após a remoção de duplicidades, restaram 599 registros para a fase de seleção, dos quais 578 foram excluídos após a análise de título e resumo. Na fase de elegibilidade, 21 artigos de texto completo foram avaliados, resultando na exclusão de 11 estudos por motivos como indisponibilidade de texto, população incorreta, conceito inadequado ou critérios de tipo de artigo. Ao final, dez estudos foram incluídos na análise. O fluxograma presente na Figura 1 apresenta síntese do processo de seleção dos artigos.

**Figura 1:** Fluxograma segundo critérios do *Preferred Reporting Items for Systematic Reviews and Meta-Analyses Extension for Scoping Reviews* segundo o JBI, Brasil, 2024

Todos os estudos incluídos na revisão (n=10) foram publicados em língua inglesa. Quanto à distribuição geográfica, os artigos abrangem pesquisadores de diversos países, incluindo Espanha, Canadá, Estados Unidos da América, Libéria, Noruega, Turquia, África do Sul, México, Colômbia e Índia, refletindo um interesse global na aplicação de IA para o enfrentamento da violência. Os métodos de pesquisa variaram entre ensaios teóricos (n=2; 20%), revisões (n=2; 20%), estudos metodológicos (n=1; 10%), método misto (n=1; 10%), análise documental (n=1; 10%), estudo transversal (n=1; 10%), estudo de avaliação (n=1; 10%) e relato de experiência (n=1; 10%). As publicações ocorreram entre 2014 e 2025, com concentração em 2024 (n=4; 40%). As sínteses detalhadas dos estudos selecionados são apresentadas no Quadro 2.

**Quadro 2:** Síntese dos materiais selecionados, São Paulo, São Paulo, Brasil, 2025

Ano	Autores/título	Objetivo	Desenho do estudo	Desfechos	Qualidade metodológica
2023	Garcia-Vergara E, Almeda N, Fernández-Navarro F, Becerra-Alonso D^(15)^ Espanha *Artificial intelligence extracts key insights from legal documents to predict intimate partner femicide*	Identificar informações relevantes em documentos legais para prever feminicídio por parceiro íntimo por meio da inteligência artificial.	Análise documental	Os modelos de *machine learning* e processamento de linguagem natural apresentaram taxa de detecção superior a 75% dos casos letalidade e não letalidade (com elevada gravidade), mostrando-se útil para predição de risco das vítimas.	Alta
2025	Abdulai AF^(16)^ Canadá *Is Generative AI Increasing the Risk for Technology-Mediated Trauma Among Vulnerable Populations?*	Examinar como as intervenções digitais em saúde alimentadas por inteligência artificial generativa podem gerar traumas emocionais entre populações vulneráveis e apresentar estratégias ​​para mitigar tais traumas no contexto de intervenções digitais em saúde.	Ensaio teórico	Conteúdos gerados por inteligência artificial, como aconselhamento de saúde ou interações automatizadas, podem inadvertidamente ativar ou reativar experiências traumáticas em indivíduos vulneráveis, como vítimas de violência. Desenvolvedores e pesquisadores de inteligência artificial devem adotar abordagens informadas sobre traumas e considerar as necessidades específicas de populações vulneráveis e pessoas violentadas ao projetar e implementar tecnologias de inteligência artificial na saúde.	Moderada
2024	Thomas A, Asnes A, Libby K, Hsiao A, Tiyyagura G^(17)^ Estados Unidos da América *Developing and Testing the Usability of a Novel Child Abuse Clinical Decision Support System: Mixed Methods Study*	Desenvolver e testar a usabilidade de um sistema de suporte à decisão clínica de casos de abuso infantil baseado em processamento de linguagem natural, conhecido como *Child Abuse Clinical Decision Support*.	Método misto	O desenho centrado no usuário e conteúdo baseado em evidências do sistema auxiliou os profissionais no reconhecimento e avaliação em tempo real de abuso físico infantil, e tem o potencial de reduzir o número de casos perdidos.	Alta
2024	Rahman R *et al*.^(18)^ Libéria *A comparative study of machine learning algorithms for predicting domestic violence vulnerability in Liberian women*	Prever a vulnerabilidade das mulheres à violência doméstica utilizando uma abordagem de *machine learning* e uso dados da Pesquisa Demográfica e de Saúde da Libéria.	Estudo de avaliação	O *Light Gradient-Boosting Machine* e o *Random Forest* foram os modelos de inteligência artificial que obtiveram maio precisão de previsão de vulnerabilidade de mulheres à violência doméstica, com acertos de 81% e 82%, especialmente a violência emocional.	Alta
2014	Hammer HL^(19)^ Noruega *Detecting Threats of Violence in Online Discussions Using Bigrams of Important Words*	Desenvolver um método automatizado para identificar ameaças de violência em discussões *online* utilizando *machine learning*.	Relato de experiência	Foram coletadas 24.840 sentenças de comentários do *YouTube*, que foram manualmente anotadas como contendo ou não ameaças violentas. Esses dados foram utilizados para treinar e testar modelos de *machine learning.* O modelo treinado apresentou uma taxa de erro de aproximadamente 10% ao classificar uma sentença violenta como não violenta, e 5% de taxa de erro em classificar sentenças não violentas como violentas. A inclusão de bigramas compostos por palavras importantes e a consideração da distância entre essas palavras na sentença resultaram no melhor desempenho de classificação.	Moderada
2024	Başaran F, Duru P^(20)^ Turquia *Determining domestic violence against women using machine learning methods: The case of Türkiye*	Determinar a frequência e os fatores de risco de violência doméstica contra mulheres aplicando métodos de *machine learning*.	Transversal	Desempenho de algoritmos *machine learning* apresentou sensibilidade entre 91% e 100% e especificidade entre 85% e 100% nos 630 casos analisados. Tempo de duração do casamento (após dez anos de união), número elevado de filhos e baixo nível de educação foram sugeridos como indicadores de risco de violência pela inteligência artificial.	Alta
2020	Hunt X *et al*.^(21)^ África do Sul *Artificial Intelligence, Big Data, and mHealth: The Frontiers of the Prevention of Violence Against Children*	Mapear as principais literaturas científicas sobre o uso da inteligência artificial para a prevenção violência contra a criança.	Revisão	A *machine learning* associada à *big data* é apontada com potencial promissor para redução da violência contra crianças, especialmente em países de baixa e média renda. Os modelos devem abarcar informações de grandes territórios (cidades inteiras) sobre preditores de violência para gerar algoritmos de prevenção e controle. Aspectos éticos, como sigilo e controle de acesso, também são apontados para a implementação de sistemas de inteligência artificial.	Moderada
2023	Cruz-Mendoza MC *et al*.^(22)^ México *Machine Learning Applied to Improve Prevention of, Response to, and Understanding of Violence Against Women*	Descrever aplicação *web* baseada em *machine learning* para coletar casos de violência por parceiro íntimo e predizer severidade.	Metodológico	O modelo de *Random Forest* se destacou como a melhor ferramenta para identificar casos de violência doméstica, atingindo taxa de acerto de 97% de acertos. O aplicativo diagnosticou os casos e gerou mapas que destacam as áreas de maior risco de violência.	Moderada
2025	Pinto-Muñoz CC *et al*.^(23)^ Colômbia *Machine Learning Applied to Gender Violence: A Systematic Mapping Study*	Identificar tendências metodológicas e mapear sistematicamente o uso de *machine learning* aplicada à violência de gênero.	Revisão	O processamento de linguagem natural e modelos de predição são as tendências metodológicas principais. Os resultados indicaram que os modelos de inteligência artificial possuem uma alta capacidade de previsão, representando uma melhoria significativa em comparação com os sistemas de avaliação de risco atualmente em uso. Há evidências de poucos estudos sobre o tema na América Latina e Caribe.	Alta
2024	Tiwari A K, Marisport A^(24)^ Índia *Leveraging Artificial Intelligence to Address Domestic Violence Against Women with Disabilities in India*	Identificar como a tecnologia de inteligência artificial pode aprimorar táticas de descoberta, prevenção da violência doméstica em mulheres com deficiência na Índia.	Ensaio teórico	Aplicativos móveis para denunciar abusos, assistentes virtuais para fornecer informações e suporte podem oferecer análises preditivas para auxiliar as autoridades de saúde e segurança na tomada de decisão. A segurança das informações das vítimas é fundamental para que não ocorra vazamento de dados que aumentem sua vulnerabilidade.	Moderada

A avaliação metodológica dos estudos demonstrou que a maioria apresentou qualidade elevada ou moderada, com delineamentos variados. Essa diversidade impossibilitou a comparação direta das amostras, mas contribuiu para um panorama abrangente sobre as múltiplas formas de aplicação da IA no enfrentamento da violência.

As populações mais abordadas foram mulheres em situação de violência doméstica, crianças vítimas de abuso e pessoas com deficiência. Alguns estudos também trataram de populações em vulnerabilidade socioeconômica ou residentes de países de baixa e média renda, onde as desigualdades tecnológicas tornaram o uso da IA ainda mais relevante.

Da análise dos achados, emergiram três grandes temas: 1) Predição e monitoramento automatizado da violência^(15,18,20,22,23)^; 2) Apoio à decisão, inovação tecnológica em saúde e proteção social^(17,21,24)^; e 3) Ética, segurança e impacto social da inteligência artificial em contextos de vulnerabilidade^(16,19)^.

Os estudos da categoria “Predição e monitoramento automatizado da violência” mencionam que os modelos de *machine learning* e processamento de linguagem natural (PLN) alcançaram alta precisão na previsão e identificação de casos de violência interpessoal, com taxas de acerto entre 75% e 97%^(15,18,20,22,23)^. As ferramentas desenvolvidas foram capazes de reconhecer padrões de risco a partir de dados legais, demográficos e comportamentais, destacando fatores como tempo de casamento prolongado, baixo nível educacional e número elevado de filhos.

Os estudos exploraram o uso de bigramas e técnicas de mineração de texto em registros judiciais e redes sociais, o que permitiu identificar ameaças de violência e gerar mapas de risco em tempo real, mostrando a capacidade da IA em reconhecer padrões linguísticos e comportamentais associados à violência interpessoal. Isso reforça o potencial da IA como instrumento de vigilância capaz de antecipar situações de perigo e apoiar políticas públicas de segurança e saúde^(15,18,20,22,23)^.

A eficácia desses modelos dependeu de bases de dados amplas, contextualizadas e culturalmente sensíveis, além de exigir validações constantes que evitassem vieses e interpretações incorretas^(18,20,23)^. A IA não se configurou como substituta da análise humana, mas como uma aliada no reconhecimento de padrões complexos e na prevenção de eventos violentos^(15,18,20,22,23)^.

Na categoria “Apoio à decisão, inovação tecnológica em saúde e proteção social”, os estudos ressaltaram o uso da IA como ferramenta de suporte ao cuidado e à tomada de decisão em contextos de violência. O desenvolvimento de aplicativos móveis e assistentes virtuais permitiu denunciar abusos, buscar informações e receber orientações de forma segura e acessível^(21,24)^. Em países com infraestrutura limitada, a combinação de *machine learning*, b*ig data* e tecnologias móveis (*Mobile Health*) mostrou potencial para reduzir a subnotificação de casos e fortalecer redes de proteção social.

Essas iniciativas aproximaram a tecnologia da prática assistencial, tornando a IA uma ferramenta de cuidado humanizado. Os estudos, entretanto, enfatizaram que o avanço dessas soluções requisitou investimentos em infraestrutura tecnológica, capacitação profissional e políticas de governança de dados que assegurassem a confidencialidade e a equidade no acesso às ferramentas.

Os estudos agrupados na categoria “Ética, segurança e impacto social da inteligência artificial em contextos de vulnerabilidade” trouxeram reflexões éticas e emocionais sobre o uso da IA em temas de violência. Observou-se que interações automatizadas, como aconselhamentos gerados por IA, puderam reativar experiências traumáticas em vítimas e sobreviventes, caso não fossem cuidadosamente desenhadas. Vazamentos de informações e uso indevido de registros geraram novas formas de vulnerabilidade, transformando o que seria uma ferramenta de proteção em fonte de risco. A criação de protocolos éticos robustos, transparência algorítmica e participação social nas etapas de desenvolvimento e implementação foi relatada como essencial^(16,19)^.

Esses aspectos reforçaram que, por trás de cada dado analisado, existiram pessoas reais, com histórias e dores singulares, e que a tecnologia só cumpriu seu papel quando conseguiu proteger e respeitar essas vidas.

## DISCUSSÃO

Apesar de as produções terem se concentrado nos Estados Unidos da América, a presença de pesquisas em várias regiões do mundo e concentradas nos últimos anos mostra que a temática é de interesse global e bastante contemporânea, e o debate gira em torno de potencialidades e também das limitações de sistemas inteligentes.

Os achados desta revisão dialogam de forma crítica e consonante com um corpo crescente de literatura científica internacional. A precisão (75%-97%) dos modelos de predição e monitoramento automatizado corrobora estudos internacionais que destacam o potencial do *machine learning* e do PLN para identificar padrões de risco complexos. Pesquisa desenvolvida nos Estados Unidos da América utilizou a mineração de texto em registros de serviços sociais, demonstrando como algoritmos podem prever recorrência de maus-tratos infantis com uma acurácia significativa, apoiando a triagem de casos^(25)^. No entanto, a ressalva presente nos resultados sobre a dependência de bases de dados “amplas, contextualizadas e culturalmente sensíveis”^(18,20,23)^ remete às advertências descritas na literatura. Estudo norte-americano evidencia como modelos preditivos em saúde priorizaram erroneamente pacientes brancos em detrimento de negros com necessidades clínicas equivalentes^(26)^, um viés que pode ser replicado em sistemas de triagem de violência, subnotificando ainda mais grupos marginalizados.

A promessa de uma vigilância preditiva eficaz também esbarra em desafios de implementação prática. Enquanto os resultados apontam para a geração de “mapas de risco em tempo real”^(15,19,22,23)^, pesquisadores australianos alertam que a expansão de tecnologias móveis de saúde, sem uma estrutura de proteção à privacidade, pode expor as vítimas a novos riscos, como o acesso não autorizado aos seus dados por parte dos agressores, situação particularmente crítica no contexto de violência doméstica^(27)^. Essa vulnerabilidade transforma dispositivos de proteção em instrumentos de risco, exigindo que o desenvolvimento tecnológico ande de mãos dadas com a segurança digital.

Pesquisadores no Reino Unido alertam ainda para o risco de “hipervigilância seletiva” em comunidades já marginalizadas, onde a presença de forças de segurança é historicamente maior e a confiança, muito menor^(28)^. Essa prática pode criminalizar a pobreza e perpetuar ciclos de intervenção traumática, em vez de canalizar recursos para apoio social e comunitário. Portanto, a eficácia técnica da IA em prever violência deve ser constantemente balanceada com uma análise crítica de seu impacto social e seu potencial para reforçar estigmas.

Os resultados apontaram que a IA como uma “aliada”, não como uma substituta, da análise humana^(15,18,20,22,23)^. Esse fator é importante, pois estudos conduzidos na Europa com *chatbots* de suporte a vítimas de violência de gênero descobriram que, embora úteis para informações básicas, essas ferramentas frequentemente falham em responder adequadamente a cenários complexos que envolvem violência psicológica ou interseccionalidade, desconsiderando nuances contextuais fundamentais para um acolhimento humanizado^(29)^.

As reflexões éticas e emocionais agrupadas na terceira categoria dos resultados^(16,19)^ constituem talvez o ponto de maior convergência com as críticas mais contundentes. A constatação de que interações automatizadas podem reativar traumas está em sintonia com estudos que questionam a capacidade dos sistemas de IA de interpretar subtextos e nuances culturais. A ênfase na necessidade de “protocolos éticos robustos e transparência algorítmica”^(16,19)^ é consequência direta do debate sobre os “sistemas de caixa preta”. Pesquisadores canadenses mencionam que a superação desses desafios exige uma abordagem de governança participativa e multissetorial, incorporando as perspectivas de vítimas, sociedade civil e especialistas em ética^(30)^. Tais pesquisadores defendem que a avaliação de impacto algorítmico deve ser um requisito obrigatório antes da implantação de qualquer sistema em contextos de vulnerabilidade^(30)^.

A constatação nos resultados sobre a necessidade de protocolos e governança encontra sinergia em análises jurídicas comparadas. Enquanto a União Europeia avança com seu *Artificial Intelligence Act*, que classifica sistemas de alto risco e impõe exigências de transparência, e o Brasil conta com a Lei Geral de Proteção de Dados, especialistas argumentam que nenhum desses marcos é suficientemente específico para abordar os riscos únicos enfrentados por vítimas de violência assistidas por IA^(31)^. A elaboração de normas setoriais, que prevejam auditorias independentes, mecanismos de contestação e a proibição de usos particularmente danosos, torna-se, portanto, uma urgência global.

Assim, os achados deste estudo refletem o estado da arte da aplicação da IA, bem como espelham os principais eixos de tensão identificados pela literatura científica global. O caminho a seguir, portanto, não reside na simples adoção tecnológica, mas na sua integração de forma crítica e regulada. A IA não se apresenta como uma panaceia, mas como uma ferramenta que, para cumprir sua promessa de proteção, deve ser continuamente avaliada, regulada e submetida à avaliação democrática, garantindo que a eficiência algorítmica nunca se sobreponha à garantia dos direitos humanos e à dignidade das vítimas.

### Limitações do estudo

Este estudo possui algumas limitações importantes. Apesar de terem sido consultadas as principais bases de dados da área da saúde e também ter sido realizada a busca de materiais cinzentos, artigos sobre o tema podem estar indexados em outras bases que não foram acessadas. Outra limitação foi a grande amplitude de métodos nas pesquisas que compuseram a amostra, levando à impossibilidade de avaliação de medidas exitosas, tanto em números absolutos quanto relativos. Destaca-se que a pouca menção nos estudos selecionados sobre algumas populações também vulneráveis, como pessoas pretas, comunidade LGBTQIA+, pessoas em situação de rua, entre outras, gera dificuldade na inferência sobre as medidas cabíveis a essas populações.

### Contribuições para as áreas da enfermagem e ciências

Apesar das limitações citadas, o estudo mostra-se altamente relevante e atual na perspectiva de que a temática da IA se mostra como uma ferramenta a mais no enfrentamento do agravo, importante em todo o mundo e com alto impacto para as populações mais vulneráveis.

## CONCLUSÕES

Esta revisão teve o objetivo de mapear as principais aplicações da IA no combate à violência interpessoal contra populações vulneráveis. Verificou-se que sistemas de *machine learning* e PLN alcançaram relevante precisão na predição e monitoramento, embora sua eficácia dependesse diretamente de bases de dados representativas e culturalmente sensíveis, sob risco de amplificarem vieses sociais. O risco de “hipervigilância seletiva” em comunidades já marginalizadas pode criminalizar a pobreza, desviar o objetivo de enfrentamento e aumentar preconceitos.

Os achados acerca de aspectos éticos indicaram que interações automatizadas puderam reativar traumas, alertando sobre o risco à privacidade, pois a falta de proteção em tecnologias móveis pode expor as vítimas a novos perigos, como o acesso não autorizado aos seus dados por agressores. A superação desses desafios exige governança participativa e multissetorial, sempre que possível. A necessidade de protocolos éticos e transparência foi apontada, bem como a afirmação de que os marcos regulatórios existentes não são suficientemente específicos. Fazem-se necessários regulamentos legais para o uso da IA, de acordo com a realidade de cada país, porém sempre respeitando os princípios de justiça, beneficência e respeito à autonomia. Estudos epidemiológicos, intervencionistas e com melhores níveis de evidência são fundamentais para avanço da temática, com aumento do incremento de outras populações altamente vulneráveis, como pessoas pretas, membros da comunidade LGBTQIA+, pessoas em situação de rua e outras minorias.

## Data Availability

Os dados de pesquisa só estão disponíveis mediante solicitação.
